# Supplementary material for: Preparation and Stabilization of High Molecular Weight Poly (acrylonitrile-co-2-methylenesuccinamic acid) for Carbon Fiber Precursor
Source: Polymers (Basel). 2021 Nov 9;13(22):3862. doi: 10.3390/polym13223862 (PMC8618359; doi:10.3390/polym13223862)
Supplement: Supplementary file 1 [file polymers-13-03862-s001.zip › polymers-1414679-supplementary.pdf]

Supporting Information

Preparation and stabilization of high molecular weight poly (acrylonitrile-*co*-2-methylenesuccinamic acid) for carbon fiber precursor

Shuxian Zhang, Yanjin Dang, Xuepeng Ni, Chunshun Yuan, Huifang Chen, Anqi Ju*

Experimental section

The viscosity average molecular weight of PAN polymer was determined by the viscosity method as follows:

(1) Firstly, 0.20 g of PAN copolymer was dissolved in 15 mL of dimethyl sulfoxide. After it was completely dissolved, using a 20 mL volumetric flask to make the volume constant at 50°C. Then we adjusted the constant temperature water bath to 50°C to test the outflow time t_0 of the dimethyl sulfoxide solvent.

(2) Secondly, PAN solution outflow time: we used a pipette to draw 10 mL of the solution and injected it into the viscometer to measure the outflow time t_1. Then 5 mL of solvent dimethyl sulfoxide was transferred to mix evenly, and measure the outflow time t_2. In the same operation, we added 10, 10, and 10 mL of solvent successively to measure the time t_3, t_4 and t_5 respectively. The formula is as following:

$$\eta_r=\frac{t}{t_0},\ \eta_{sp}=\eta_r\text{-}1$$

Where η_r is the relative viscosity, and η_{sp} is the specific viscosity. Calculate a series of $In(\eta_r/c)$, η_{sp}/c, and then make the curve of $In(\eta_r/c)$, η_{sp}/c versus concentration c, the intrinsic viscosity $[\eta]$ is calculated by linear extrapolation of c too zero.

$$[\eta]=2.83*10^{-2}{M_\eta}^{0.758}$$

Table S1. the value of intrinsic viscosity by the viscosity method

AN/MLA(wt/wt)	99/1	98/2	97/3	96/4	95/5
$[\eta]$(dL/g)	6.71	6.21	5.67	4.61	3.55

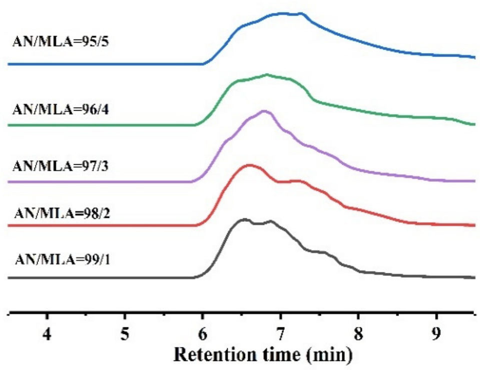

Figure S1. GPC trace of PAN(AN-co-MLA) with different mass feed monomer ratios.

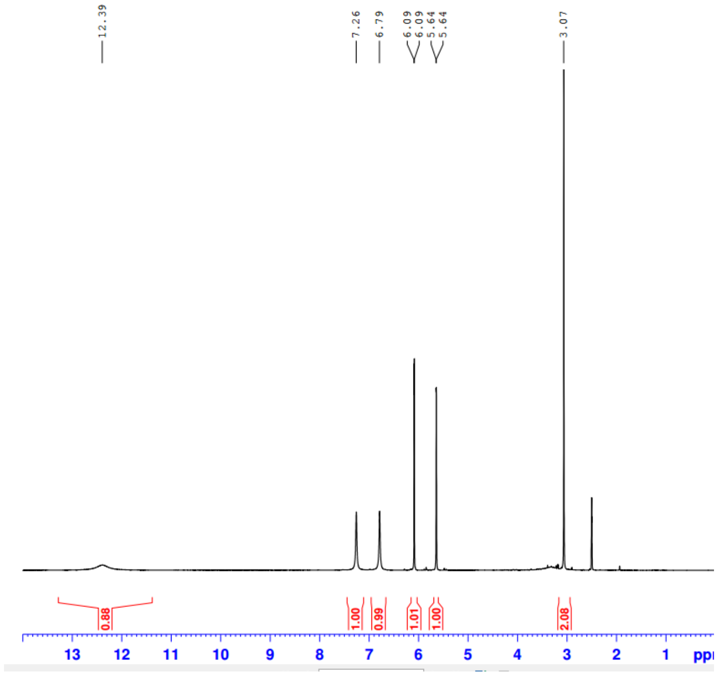

Figure S2. ^{1}H NMR spectrum of MLA

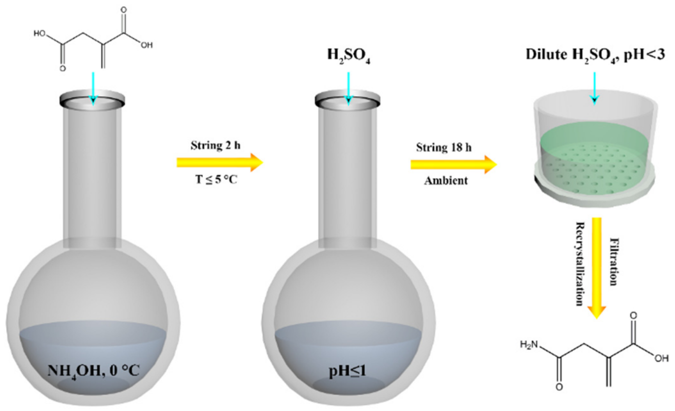

Scheme S1. Synthesis process of MLA for P(AN-*co*-MLA)

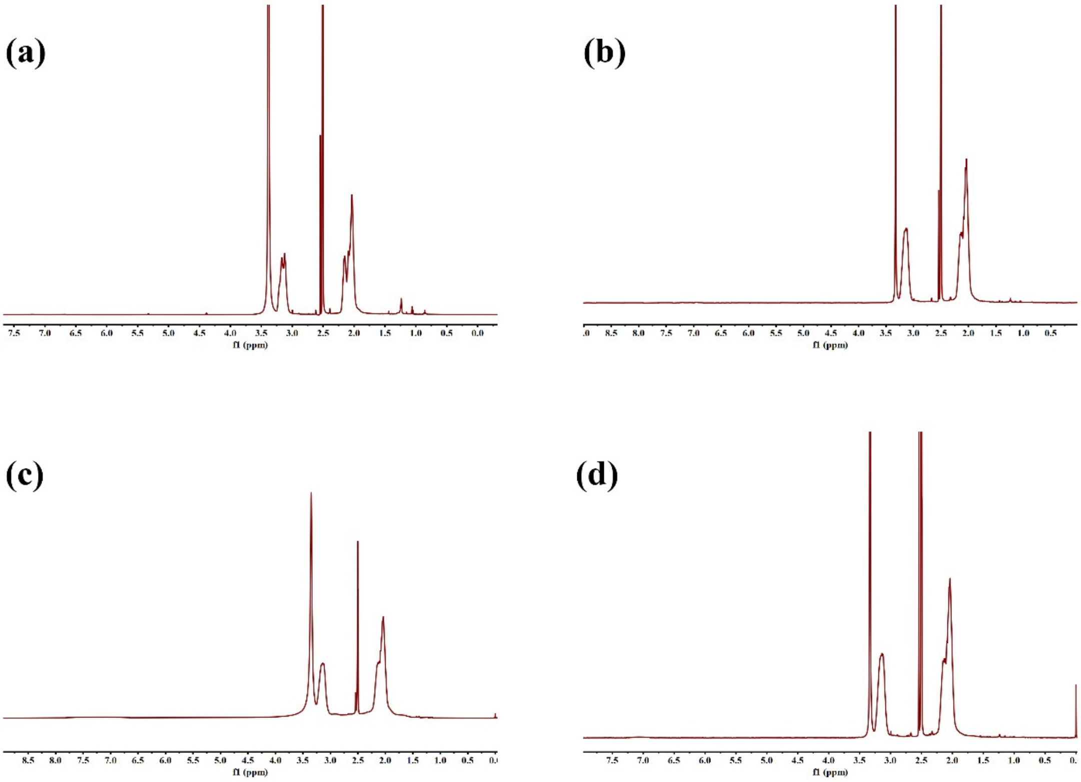

Figure S3. ^{1}H NMR spectrum of PAN(AN-co-MLA) with different mass feed monomer ratios (wt/wt): (a) AN/MLA=99/1, (b) AN/MLA=98/2, (b) AN/MLA=96/4, (b) AN/MLA=97/3.
